# Resolving Coffee Waste and Water Pollution—A Study on KOH-Activated Coffee Grounds for Organophosphorus Xenobiotics Remediation

**DOI:** 10.3390/jox14030070

**Published:** 2024-09-10

**Authors:** Vedran Milanković, Tamara Tasić, Igor A. Pašti, Tamara Lazarević-Pašti

**Affiliations:** 1 VINCA Institute of Nuclear Sciences-National Institute of the Republic of Serbia, University of Belgrade, Mike Petrovica Alasa 12-4, 11000 Belgrade, Serbia; vedran.milankovic@vin.bg.ac.rs (V.M.); tamara.tasic@vin.bg.ac.rs (T.T.); 2 Faculty of Physical Chemistry, University of Belgrade, Studentski Trg 12-16, 11158 Belgrade, Serbia; igor@ffh.bg.ac.rs

**Keywords:** pesticides, malathion, chlorpyrifos, adsorption, AChE, neurotoxicity, wastewater, food biowaste, environmental risk

## Abstract

This study investigates using KOH-activated coffee grounds (KACGs) as an effective adsorbent for removing organophosphorus xenobiotics malathion and chlorpyrifos from water. Malathion and chlorpyrifos, widely used as pesticides, pose significant health risks due to their neurotoxic effects and environmental persistence. Spent coffee grounds, abundant biowaste from coffee production, are chemically activated with KOH to enhance their adsorptive capacity without thermal treatment. This offers a sustainable solution for biowaste management and water remediation. Adsorption kinetics indicating rapid initial adsorption with high affinity were observed, particularly for chlorpyrifos. Isotherm studies confirmed favorable adsorption conditions, with higher maximum adsorption capacities for chlorpyrifos compared to malathion (15.0 ± 0.1 mg g^−1^ for malathion and 22.3 ± 0.1 mg g^−1^ for chlorpyrifos), highlighting its potential in mitigating water pollution. Thermodynamic analysis suggested the adsorption process was spontaneous but with the opposite behavior for the investigated pesticides. Malathion interacts with KACGs via dipole–dipole and dispersion forces, while chlorpyrifos through π–π stacking with aromatic groups. The reduction in neurotoxic risks associated with pesticide exposure is also shown, indicating that no more toxic products were formed during the remediation. This research contributes to sustainable development goals by repurposing biowaste and addressing water pollution challenges through innovative adsorbent materials.

## 1. Introduction

**Figure 1:**
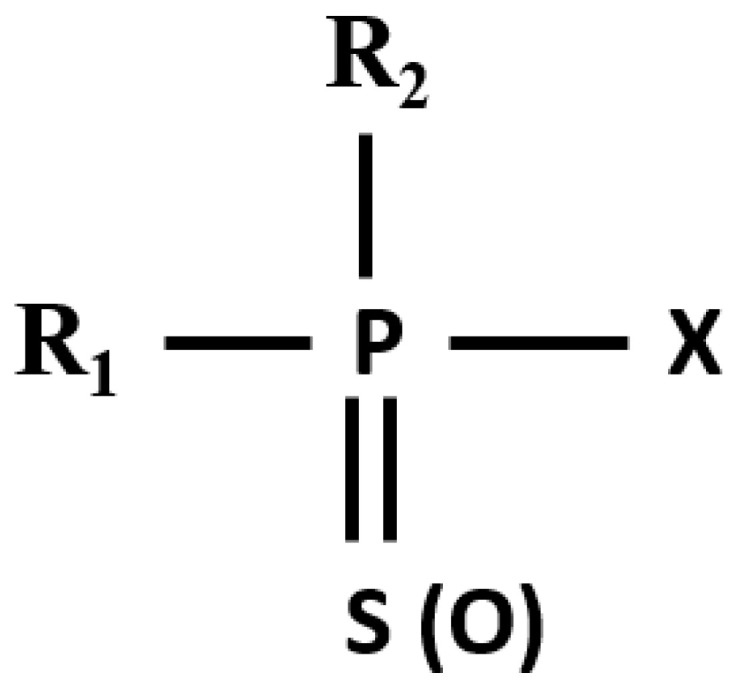
The general structure of organophosphate pesticides.

Pesticides are harmful xenobiotics that pose significant risks to human health and the environment. Among these, organophosphates (OPs), whose general structure is given in Figure 1, are widely used in agricultural, commercial, and residential settings, leading to everyday exposure for the general population [1]. OPs are a class of chemicals characterized by their phosphorus-containing molecular structures, which include various functional groups that influence their chemical behavior and toxicity. Two significant functional groups in OPs are the =S(O) group (a sulfur or oxygen double bond) and the -X group, where X typically represents a leaving group such as Cl, F, or an alkoxy group (OR). The =S(O) group plays a crucial role in the compound’s reactivity and its interaction with biological targets, such as the enzyme acetylcholinesterase (AChE). The -X group, on the other hand, determines the compound’s stability, solubility, and ease of hydrolysis, impacting its persistence in the environment and its ability to penetrate biological membranes [2]. These compounds are toxic to humans and animals primarily because they inhibit the enzyme AChE, which is crucial for terminating nerve impulse transmission by hydrolyzing the neurotransmitter acetylcholine. The inhibition of AChE results in the accumulation of acetylcholine, causing hyperstimulation of nicotinic and muscarinic receptors and disrupting normal neurotransmission [3,4]. The toxicity of OPs extends beyond acute effects to include chronic health issues such as neurological, respiratory, and reproductive problems [5,6].

**Figure 2:**
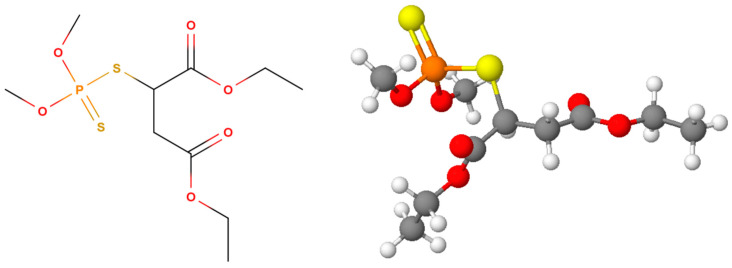
Structure of malathion (carbon atoms are shown in grey, hydrogen in white, oxygen in red, sulfur in yellow, and phosphorus in orange).

Malathion (O,O-dimethyl-S-(1,2-dicarbethoxyethyl) phosphorodithioate; MLT) is a xenobiotic organophosphate insecticide extensively used in agriculture, public health, and veterinary medicine to manage pests such as mosquitoes, fruit and vegetable pests, and ectoparasites on livestock [7]. It is an aliphatic OP with molecular formula C_10_H_19_O_6_PS_2_ and structure as shown in Figure 2 [8]. It is classified as moderately toxic and can biotransform into its more toxic form, malaoxon, significantly increasing its toxicity [9]. Although effective in pest control, MLT poses significant health risks, including neurological, respiratory, and reproductive problems, acute symptoms like headaches, dizziness, and nausea, and chronic effects such as endocrine disruption and immunotoxicity [10]. Continuous exposure to MLT can also lead to elevated antibody levels, indicative of autoimmune responses [11].

**Figure 3:**
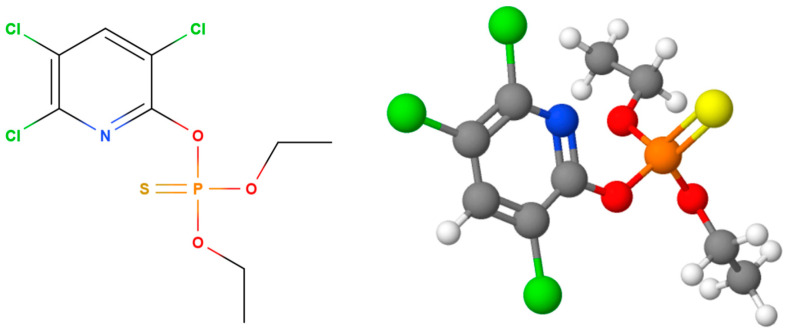
Structure of chlorpyrifos (carbon atoms are shown in grey, hydrogen in white, oxygen in red, sulfur in yellow, phosphorus in orange, chlorine in green, and nitrogen in blue).

Chlorpyrifos (O,O-diethyl-O-(3,5,6-trichloro-2-pyridyl) phosphorothioate; CHP) is a xenobiotic organophosphate insecticide widely utilized in agriculture, households, and veterinary medicine to manage pests such as cockroaches, fleas, termites, and ticks, as well as to protect crops from various insects [12]. CHP has the molecular formula C_9_H_11_Cl_3_NO_3_PS and structure shown in Figure 3 [13]. Classified as a highly toxic pesticide, CHP can be biotransformed in the liver into chlorpyrifos-oxon, a metabolite up to 180 times more toxic than the parent compound, with an LD50 in rats of 95–270 mg/kg [14]. Despite its effectiveness in pest control, CHP poses significant health risks, including psychological, endocrine, hematological, respiratory, and reproductive issues. It is particularly harmful to fetuses and children at low exposure levels, causing neurological damage, and can lead to acute poisoning in cases of high exposure. Continuous exposure to CHP has been linked to elevated levels of specific antibodies, indicating autoimmune diseases, and there is a strong association between chronic illnesses related to autoimmune disorders and chlorpyrifos exposure [15,16].

As xenobiotics, MLT and CHP’s presence in biological systems highlights their potential for causing adverse health effects upon exposure. Therefore, addressing the environmental and health impacts of these compounds is critical. Using biowaste materials in environmental protection offers a sustainable solution to managing waste while benefiting the ecosystem [17]. Moreover, repurposing biowaste addresses the environmental problem of waste disposal and promotes a circular economy by turning waste into resources [18,19].

As already mentioned, MLT and CHP are organophosphorus pesticides with significant neurotoxic effects, primarily inhibiting the enzyme AChE, which leads to the accumulation of acetylcholine in nerve synapses, causing a range of toxic effects, including neurological disorders, respiratory issues, and even death in severe cases. Prolonged exposure to low levels of these pesticides has also been linked to developmental and reproductive problems, as well as potential carcinogenic effects [10,15]. Environmentally, MLT and CHP are persistent, accumulating in soils and sediments, eventually leaching into groundwater and surface waters, where they bioaccumulate in aquatic organisms and pose risks up the food chain, including to humans. These pesticides often enter water bodies through agricultural runoff, improper disposal, and industrial discharges, leading to their frequent detection in rivers, lakes, and groundwater at varying concentrations that often exceed safety thresholds [11]. The removal of MLT and CHP from water is challenging due to their chemical stability and low water solubility, with conventional treatment processes like coagulation, flocculation, sedimentation, and filtration being insufficient. While advanced oxidation processes (AOPs) and adsorption onto activated carbon have been explored, they are often costly, energy-intensive, and may produce harmful byproducts [20].

Spent coffee grounds (SCGs), a byproduct of coffee production and consumption, are increasingly recognized as valuable biowaste materials due to their renewable, cost-effective, and safe nature, making them suitable for various applications. Recent research indicates that approximately 10.5 million metric tons of coffee are produced annually in about 50 countries, with Brazil and Vietnam being the most significant suppliers. The rising volume of SCGs poses environmental and logistical challenges due to increased coffee consumption worldwide [21].

Improper disposal of SCGs can have significant environmental impacts. The primary environmental damages caused by coffee grounds are air, water, and land pollution [22]. When exposed to the environment, coffee grounds release nitrogen and carbon dioxide. Nitrogen reacts with oxygen to form smog and ozone, both detrimental to the environment, while carbon dioxide, a greenhouse gas, contributes to climate change. SCGs can also cause water pollution by introducing chemicals, increasing acidity levels, and promoting harmful algal blooms and bacteria, negatively affecting aquatic life and human health. SCGs can attract pests on land and disrupt plant growth due to their organic and inorganic compounds [23].

To mitigate the environmental impact of coffee grounds, they should be composted, reused, or recycled. Proper disposal can minimize their environmental harm. SCGs can serve as a precursor for biochar production due to their porosity, surface area, and rich organic composition, making them excellent candidates for synthesizing adsorbents to remove pollutants from water [24,25,26]. Their abundance and availability make SCGs a sustainable and cost-effective precursor for adsorbent synthesis, contributing to circular economy principles and waste valorization by reducing environmental pollutants and waste disposal burdens [27].

The chemical activation of biowaste enhances its adsorptive properties, making it more effective for environmental remediation and energy storage. This process involves treating biowaste with chemical agents before or during high-temperature carbonization, developing internal pore structures, and introducing functional groups with specific chemical properties. Common chemical activators include alkaline, acidic, neutral, and self-activating agents. KOH and H_3_PO_4_ are particularly effective and extensively studied due to their availability, cost-effectiveness, and ability to generate specific pore types [28,29,30].

A few studies have explored biomass-derived carbon materials for OP remediation. To date, none of them has been proven to reduce AChE activity inhibition, which would indicate that during the process of remediation, OPs did not transform into their more toxic byproducts. Various carbonization and activation processes have yielded different adsorption capacities, indicating that activation methods significantly influence performance. For example, sugarcane bagasse carbonized at 450 °C showed an adsorption capacity of 3.20 mg g^−1^ for CHP [29,30,31]. In contrast, tobacco-derived carbon materials activated with ZnCl_2_ and NaOH showed adsorption capacities varying in the 0.463–1.602 mg g^−1^ range for CHP remediation [32]. Bamboo and coconut shell-derived carbon materials activated with steam showed moderate capacities (0.588 mg g^−1^ for bamboo and 0.500 mg g^−1^ for coconut shell-derived activated carbon), highlighting the influence of activation methods [33]. Fig and plum pomace-derived materials activated by gamma irradiation were investigated for CHP and MLT adsorption. The q_max_ values reported for CHP adsorption were both 0.495 mg g^−1^, and for MLT, 0.625 mg g^−1^ and 1.067 mg g^−1^, respectively, demonstrating that gamma irradiation can be a useful activation method, though it might not significantly outperform other activation techniques in terms of capacity [34,35]. In our previous study, we demonstrated that 1g of raw SCGs can adsorb up to 7.16 mg of MLT and 7.00 mg of CHP and, therefore, slightly reduce the inhibition of AChE activity (21% reduction after MLT and 9% reduction after CHP adsorption) [36].

SCGs have been studied for their ability to adsorb a wide range of pollutants from water, leveraging their natural functional groups (hydroxyl, carboxyl, and amine) and porous structure. SCGs effectively remove heavy metals like lead, cadmium, copper, and chromium, binding these ions to significantly reduce their concentrations. They are also successful in adsorbing synthetic dyes from industrial wastewater, with removal efficiencies exceeding 80%. SCGs can adsorb organic pollutants, such as phenols, pesticides, and pharmaceuticals, due to their hydrophobic nature and surface chemistry. Additionally, SCGs help in capturing excess nutrients like ammonium and phosphate, reducing nutrient pollution in water bodies [37].

The novelty of this research lies in its innovative use of SCGs, a common biowaste treated solely with KOH without thermal activation, as an efficient adsorbent for OP removal from water, with a focus on reducing the neurotoxic effects of these OPs and preventing the formation of more toxic products. By eliminating the need for expensive and energy-intensive thermal treatments, the process becomes more cost-effective and accessible, promoting sustainability and simplifying the adsorbent preparation. Additionally, repurposing SCGs aligns with the principles of a circular economy, reducing environmental impact by converting waste into valuable resources and minimizing greenhouse gas emissions from decomposing coffee grounds in landfills. This approach addresses the dual issues of biowaste disposal and water pollution and offers a versatile solution for environmental remediation.

This research supports and advances the UN’s Sustainable Development Goals, SDG 6 (Clean water and sanitation), in particular, by remediating OPs, and SDG 12 (Responsible consumption and production) and SDG 13 (Climate action) by using climate-harmful biowaste (SCGs) in the production of carbon materials [38].

## 2. Materials and Methods

### 2.1. Material Preparation and Physicochemical Characterization of KACGs

Coffee purchased locally, which consisted of 80% Arabica and 20% Robusta, was brewed with boiling water and allowed to cool at room temperature for 2 h. The brewed coffee grounds were then separated using filtration and left to air-dry at room temperature for 24 h. Subsequently, the SCGs were dried in an oven at 80 °C for 1 h to remove any remaining moisture. To obtain KOH-activated coffee grounds (KACGs), the SCGs were then treated with KOH (c = 1 mol dm^−3^) in the weight ratio SCGs:KOH = 2:1. The treatment was conducted at room temperature (22 °C), and the SCGs were impregnated with KOH for 60 min. After the treatment, the material was washed with 50 mL of 0.1 mol dm^−3^ NaOH, 50 mL of 0.1 mol dm^−3^ HCl, and 50 mL of deionized water, and finally suspended in 50 mL of 50% ethanol to obtain a stock suspension with mass concentration of 2 mg mL^−1^ and resulting pH = 7.

The morphology and elemental composition of the sample were examined using a PhenomProX scanning electron microscope (SEM) obtained from Thermo Fisher Scientific, Waltham, MA, USA. This analysis was further enhanced by incorporating energy-dispersive X-ray analysis (EDX), which provided detailed insights into the elemental distribution and structural characteristics of the samples at the microscale.

A Fourier-transform infrared (FTIR) spectrum was obtained using a Nicolet iS20 FT-IR spectrophotometer (Thermo Fisher Scientific, Waltham, MA, USA). The spectral data was acquired over a range of wavenumbers from 4000 to 400 cm^−1^, with each measurement comprising 64 scans at a resolution of 4 cm^−1^. This analysis provided comprehensive information about the functional groups and molecular bonds within the sample.

### 2.2. Adsorption Experiments

The materials’ efficiency for removing selected OPs from aqueous solutions was investigated. MLT and CHP stock solutions (Pestinal, Sigma-Aldrich, Soborg, Denmark) were prepared at concentrations ranging from 1 × 10^−5^ to 1 × 10^−3^ mol dm^−3^. The resulting pesticide solutions and material suspensions were mixed in a 1:1 ratio, further diluting them to final adsorbent concentrations of 1 g dm^−3^ and pesticide concentrations ranging from 5 × 10^−6^ mol dm^−3^ to 5 × 10^−4^ mol dm^−3^. The mixtures were left to stir in an orbital shaker (Orbital Shaker Incubator Grant-bio ES-20, Grant-Bio, Cambridgeshire, UK) for the desired period at the appropriate temperature. After incubation, the suspensions were filtered through a nylon filter (pore size 0.22 µm, diameter 13 mm, KX Syringe Filter, Kinesis, Cole Parmer, St. Neots, UK) and prepared for chromatographic analysis. The concentration of adsorbed pesticide was determined from the difference between the initial concentration and the concentration of the remaining pesticide after adsorption. Control experiments were conducted in the same manner but without the carbon adsorbents to avoid the influence of potential pesticide degradation within the timeframe of the described experiments. To examine the influence of different working parameters and find the optimal conditions for the process, the adsorption temperature, the initial concentration of the pesticide, and the contact time were varied.

To investigate the kinetic parameters of the adsorption process, a suspension of the adsorbents at a final concentration of 1 mg mL^−1^ was incubated with MLT and CHP at a final concentration of 5 × 10^−5^ mol dm^−3^ for various time intervals ranging from 1 min to 24 h at 25 °C. The obtained experimental data of CHP and MLT adsorption onto the investigated adsorbents were used to determine the equilibrium time and were analyzed using nonlinear models: pseudo-first-order (PFO) (Equation (1)), pseudo-second-order (PSO) (Equation (2)), Elovich (Equation (3)), and intraparticle diffusion (IPD) (Equation (4)) kinetic models [39].

(1) $$q_{t}=q_{e}(1-e^{-k_{1}t})$$

(2) $$q_{t}=\frac{q_{e}^{2}k_{2}t}{1+q_{e}k_{2}t}$$

(3) $$q_{t}=\frac{1}{\beta }(1+\alpha \beta t)$$

(4) $$q_{t}=k_{id}t^{0.5}+C$$

In the Lagergren PFO model, q_t_ (mg g^−1^) is the amount of adsorbate adsorbed at time t (min), q_e_ (mg g^−1^) is the equilibrium adsorption capacity, and k_1_ (min^−1^) is the rate constant. The PSO kinetic model uses q_t_, q_e_, and k_2_ (g mg^−1^ min^−1^) as the rate constant. The Elovich kinetic model involves q_t_, the initial adsorption rate α (mg g^−1^ min^−1^), and the desorption constant β (g mg^−1^). In the IPD kinetic model, q_t_, k_id_ (mg g^−1^ min^−0.5^), t, and the boundary layer thickness C (mg g^−1^) are used [39].

Additionally, to further examine the adsorption process, 1 mg mL^−1^ of the adsorbents was incubated with MLT and CHP in a final concentration range of 5 × 10^−6^ to 5 × 10^−4^ mol dm^−3^ at temperatures of 25, 30, and 35 °C for the time determined to be the equilibrium time. The resulting data were fitted to nonlinear isotherm models, including Freundlich (Equation (5)), Langmuir (Equation (6)), Temkin (Equation (7)), and Dubinin–Radushkevich (Equation (8)) [40].

(5) $$q_{e}=K_{F}c_{e}^{\frac{1}{n}}$$

(6) $$q_{e}=\frac{q_{max}K_{L}c_{e}}{1+K_{L}c_{e}}$$

(7) $$q_{e}=\frac{RT}{b_{T}}lnK_{T}c_{e}$$

(8) $$q_{e}=q_{DR}e^{-K_{DR}\varepsilon^{2}}$$

In the Freundlich isotherm model, q_e_ (mg g^−1^) represents the amount of adsorbate on the adsorbent surface at equilibrium concentration ce (mg dm^−3^), K_F_ is the Freundlich adsorption coefficient ((dm^3^ mg^−1^)^1/n^), and n is the adsorption intensity. The Langmuir isotherm model uses q_e_, c_e_, q_max_ (mg g^−1^) as the maximum adsorption capacity of the monolayer, and K_L_ (dm^3^ mg^−1^) as the Langmuir constant indicating the affinity of the adsorbate for the adsorbent. The Temkin isotherm model involves q_e_, c_e_, b_T_ (J g mol^−1^ mg^−1^) as the Temkin constant related to adsorption energy, and K_T_ (dm^3^ mg^−1^) as the equilibrium binding constant. The Dubinin–Radushkevich isotherm model includes q_e_, q_DR_ (mg g^−1^) as the theoretical isotherm saturation capacity, K_DR_ (mol^2^ J^−2^) as the Dubinin–Radushkevich constant, and the Polanyi potential calculated using:

(9) $$\varepsilon =RTln(1+\frac{1}{c_{e}})$$

Additionally, the mean free energy E (J mol^−1^) can be derived from K_DR_ using [40]:

(10) $$E=\frac{1}{\sqrt{2K_{DR}}}$$

Thermodynamic parameters, including changes in enthalpy (ΔH), entropy (ΔS), and Gibbs free energy (ΔG), provide insights into the nature of adsorption and the spontaneity of the process. They can be calculated using experimental data obtained at different temperatures with the following equations [41]:

(11) $$lnK_{dist}^{0}=-\frac{\Delta H^{0}}{RT}+\frac{\Delta S^{0}}{R}$$

(12) $$K_{dist}^{0}=\frac{q_{e}}{C_{e}}\times \frac{C^{0}}{q^{0}}$$

The values of ΔH^0^ and ΔS^0^ can be determined as the intercept and the slope of the plot of the Van’t Hoff equation, respectively (11), where $\Delta G^{0}=-RTlnK_{dist}^{0}$. The standard distribution coefficient was calculated using Equation (12). To make it dimensionless, $\frac{q_{e}}{C_{e}}$ was multiplied with the C^0^ and q^0^ values, representing the standard state for the contaminant in the solution (1 mol dm^−3^) and the adsorbed state (1 mol kg^−1^). Gibbs free energy was then calculated using the Gibbs–Helmholtz Equation (13) [41].

(13) $$\Delta G^{0}=\Delta H^{0}-T\Delta S^{0}$$

### 2.3. Determining the Concentration of OPs Using Ultra-Performance Liquid Chromatography

**Figure 4:**
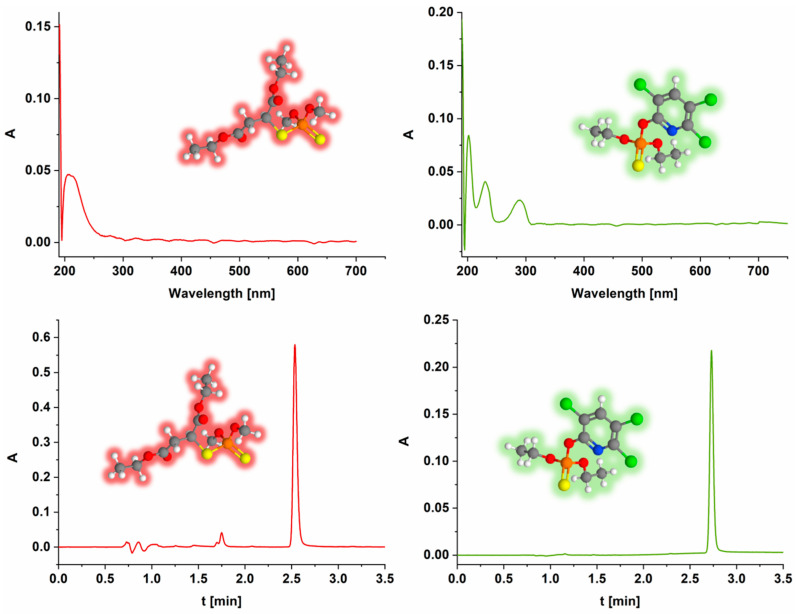
UV–VIS spectra (**top row**) and chromatograms (**bottom row**) of MLT (**left**) and CHP (**right**). Carbon atoms are shown in grey, hydrogen in white, oxygen in red, sulfur in yellow, phosphorus in orange, chlorine in green, and nitrogen in blue.

The concentration of OPs was determined using ultra-performance liquid chromatography (UPLC). A Waters ACQUITY UPLC with a PDA detector (Waters GmbH, Eschborn, Germany) was used to determine the pesticide concentration. A BEH C18 column was used under isocratic conditions: mobile phase A—10% acetonitrile in water and B—pure acetonitrile. The injected volume was 5 μL, while the flow rate was 0.2 mL min^−1^ in all the cases. The mobile phase in the case of the MLT consisted of 40% A and 60% B, while in the case of CHP, it consisted of 20% A and 80% B for individual detection. The retention time for the MLT under these conditions was 2.5 min, while it was 2.7 min for the CHP. Both pesticides were detected at a wavelength of 200 nm. All the experiments were performed in triplicate, and the deviations were calculated as the largest deviation of the experimental from the mean. The UV–VIS spectra and chromatograms of MLT and CHP are shown in Figure 4. The control experiments were performed identically but without the presence of the material.

### 2.4. Assessment of AChE Activity Inhibition

The physiological effects of the treated solutions were analyzed through AChE inhibition measurements using a modified Ellman’s procedure [42]. Commercially purified AChE from an electric eel at a concentration of 2.5 IU was exposed to the organophosphate solutions in a 50 mM phosphate buffer at pH 8.0 and 37 °C in a final volume of 0.650 mL. The enzymatic reaction was initiated by combining AChI with DTNB as the chromogenic reagent. The reaction was allowed for 8 min before being stopped with 10% sodium dodecyl sulfate (SDS). Thiocholine, the reaction product, reacts with DTNB to form 5-thio-2-nitrobenzoate, whose optical absorption was measured at 412 nm using UV–VIS Perkin Elmer, Lambda 35 spectrophotometer (Perkin Elmer, Traiskirchen, Austria). The enzyme concentration was maintained at a level that produced an optimal spectrophotometric signal. The AChE from electric eel, AChI, and DTNB were sourced from Sigma-Aldrich in St. Louis, MO, USA. Potassium hydrogen phosphate (K_2_HPO_4_·3H_2_O) and acetonitrile were acquired from Merck KgaA in Darmstadt, Germany.

The physiological effects were quantified as AChE inhibition, calculated as follows:

(14) $$I=100\times \frac{A_{0}-A}{A_{0}}$$

where A_0_ and A stand for the AChE activity in the absence of OP and the one measured after exposure to a given OP.

## 3. Results and Discussion

### 3.1. Characterization of KACGs

**Figure 5:**
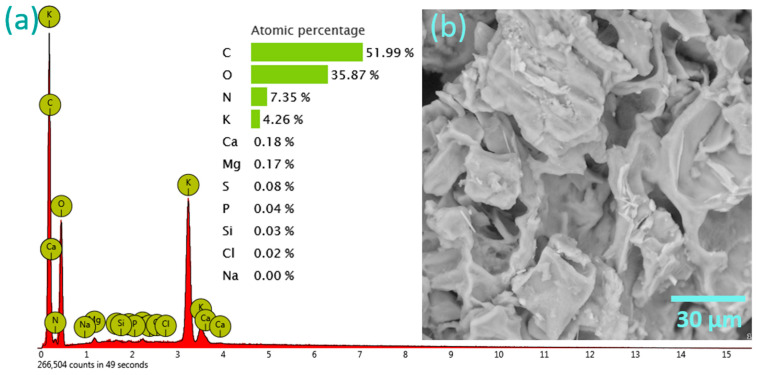
(**a**) EDX spectrum and elemental composition in at. % of KACGs; (**b**) SEM micrograph of KACGs (magnification 2000×).

SEM micrography (Figure 5b) revealed a plate-like form of the material, while elemental analysis using EDX (Figure 5a) revealed a dominant presence of C, O, and N, of 51.99 at. %, 35.87 at. %, and 7.35 at. %, respectively. In addition to these elements, as expected, K was also detected, constituting 4.26 at. % of the material.

The significant presence of K, as well as the increase in O content compared to the SCGs with a subsequent decrease in C, is attributed to the activation process with KOH, which introduces potassium ions and hydroxyl functional groups into the carbon structure. The heterogeneous surface observed post-activation can be linked to the effect of KOH. The transformation from the sponge-like structure of SCGs (Figure S1) [36] to a plate-like structure indicates a reorganization of the carbon matrix, likely enhancing the material’s mechanical stability and adsorption properties.

While it may be intuitively assumed that a sponge-like structure would provide a larger surface area and thus better adsorption properties, our results indicate that the reorganization into a plate-like structure post-KOH activation actually enhanced the adsorption efficiency, likely due to the improved mechanical stability and specific interactions with the adsorbates.

**Figure 6:**
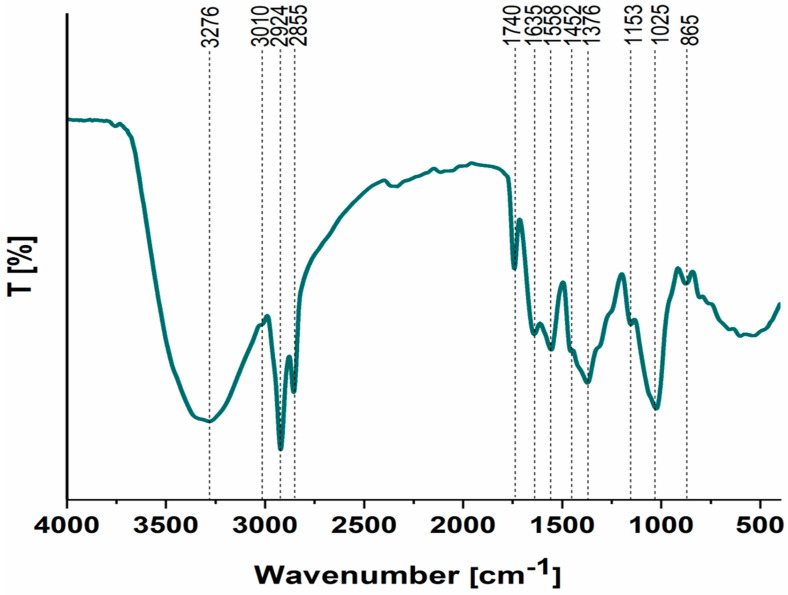
FTIR spectrum of KACGs.

The FTIR spectrum of the KACGs (Figure 6) shows almost identical vibrations as a precursor (Figure S2) [36], with some bands moving towards the lower wavenumber, indicating the chemical interaction between KOH and SCGs, and a low-intensity band at 865 cm^−1^ appearing. The broad band at 3276 cm^−1^ is attributed to the stretching of O-H groups due to hydrogen bonding. The bands at 3010 cm^−1^, 2924 cm^−1^, and 2855 cm^−1^ indicate the presence of C-H bonds, with carbon being sp^2^ and sp^3^ hybridized, respectively [43]. The sharp band at 1740 cm^−1^ corresponds to a C=O stretch vibration, while the band at 1635 cm^−1^ is attributed to a C=N stretch vibration [44]. A condensed aromatic system is assumed as the bands at 1558 cm^−1^ and 1452 cm^−1^ represent aromatic skeletal stretching and aromatic vibrations coupled with aromatic C-H in-plane vibrations, respectively. The band at 1376 cm^−1^ is assigned to aliphatic C-H stretch vibration. Deformation vibrations of C-N and C-O in secondary alcohols and C-N are indicated by the bands at 1153 cm^−1^ and 1025 cm^−1^ [45]. Finally, the band at 1025 cm^−1^ is attributed to the coupling of aromatic C-H in-plane deformation vibrations and C-O stretch vibrations in primary alcohols [46]. The new band at 865 cm^−1^ is attributed to a β-linkage in cellulose [47]. This band indicates that the activation process has introduced or preserved certain structural features of cellulose, which could impact the material’s mechanical properties and interaction with various adsorbates.

### 3.2. Kinetic Studies of MLT and CHP Adsorption onto KACGs

**Figure 7:**
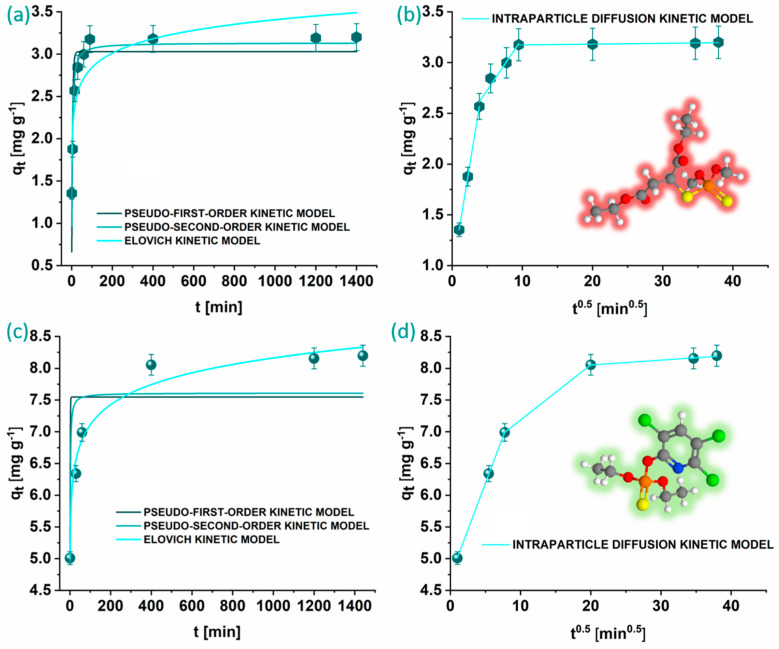
Graphical representations of kinetic models for MLT (**a**,**b**) and CHP (**c**,**d**) adsorption onto KACGs. Carbon atoms are shown in grey, hydrogen in white, oxygen in red, sulfur in yellow, phosphorus in orange, chlorine in green, and nitrogen in blue.

Equilibrium between the KACGs and MLT is achieved after 90 min, while equilibrium for the adsorption of CHP is reached after 400 min, which can clearly be seen in the graphical representations in Figure 7. The obtained parameters are shown in Table 1. Both the PFO and PSO kinetic models fail to adequately describe the adsorption of CHP onto KACGs, as evidenced by the very low R^2^ values, while PSO can be used to describe the MLT adsorption onto the KACGs. Although the rate constants k_1_ and k_2_ are higher for the adsorption of CHP, this does not align with the experimental observations, and therefore these results will not be further considered. The Elovich kinetic model, on the other hand, provided a good fit with the experimental data and indicated a high initial adsorption rate of pesticides onto the KACGs, particularly for CHP, and very low desorption rates. This suggests that the initial adsorption process is rapid and efficient, but the OPs are not easily desorbed once adsorbed. The IPD kinetic model showed that the adsorption of both OPs occurs through three separate stages. The initial rapid phase is explained by the movement of MLT and CHP molecules through the solution towards the outer surface of the KACGs. The subsequent phase involves intraparticle diffusion, where MLT and CHP molecules enter the pores of the KACGs. The third phase observed with CHP represents the equilibrium stage [48]. Throughout these stages, a gradual decrease in the k_id_ values is observed, while the C value increases until equilibrium of the adsorption process is reached.

### 3.3. Isotherm Studies for MLT and CHP Adsorption onto KACGs

**Figure 8:**
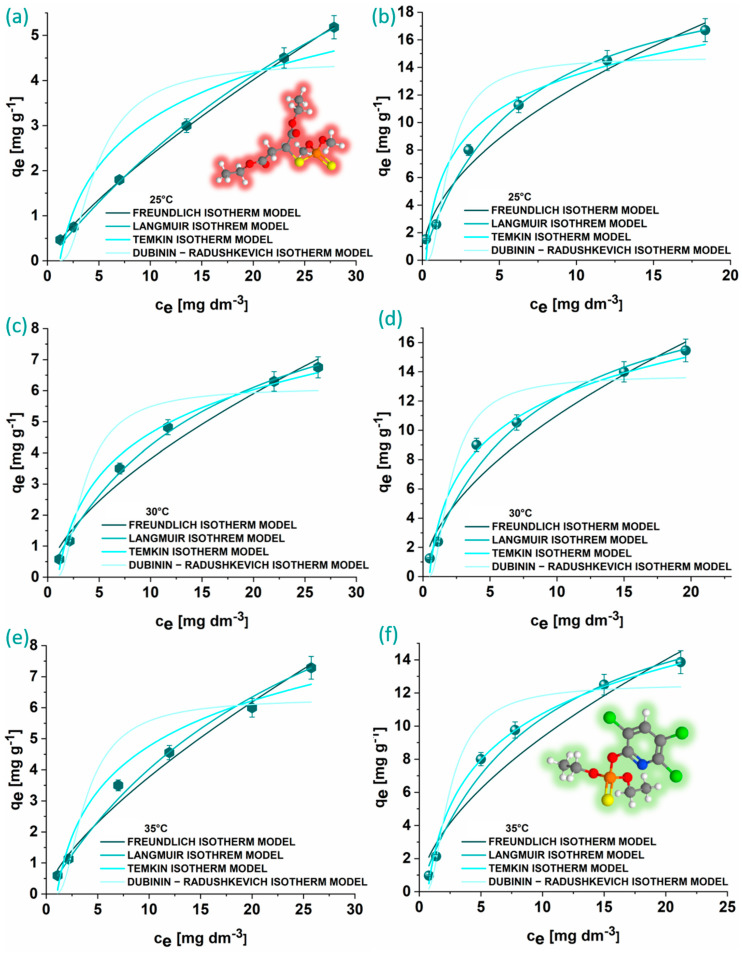
Graphical representations of isotherm models for MLT (**a**,**c**,**e**) and CHP (**b**,**d**,**f**) adsorption onto KACGs (1 mg mL^−1^) at 25 °C (**a**,**b**), 30 °C (**c**,**d**), and 35 °C (**e**,**f**).

The graphical representations of the experimental data for MLT and CHP adsorption onto KACGs, along with the corresponding isotherm model fits, are shown in Figure 8, and the calculated parameters, including R^2^ and χ^2^, are provided in Table 2.

The Freundlich isotherm shows that the adsorption processes are favorable. This is indicated by the Freundlich constant n, which is above 1 for both OPs, suggesting beneficial adsorption conditions. Notably, the n values indicate a more favorable adsorption scenario for CHP, implying a higher affinity of this OP towards KACGs compared to MLT. The q_max_ values obtained from the Langmuir isotherm model support this, as the values for CHP adsorption are higher than MLT adsorption. It can be noticed that q_max_ gradually increases for MLT and decreases for CHP adsorption onto KACGs with the increase in temperature. Therefore, the highest adsorption capacity for the MLT obtained is at 35 °C (15.0 ± 0.1 mg g^−1^), while for the CHP, it is at 25 °C (22.3 ± 0.1 mg g^−1^). The Temkin isotherm indicated a significantly stronger CHP binding affinity to the KACGs than MLT. The Temkin constant K_T_ values were up to ten times higher for CHP, highlighting its stronger interaction with the adsorbent. Further supporting these findings, the Dubinin–Radushkevich isotherm model, used to distinguish between physisorption and chemisorption, revealed that the mean free energy of adsorption for CHP is consistently higher than that for MLT across all the examined temperatures. However, since all the derived values are below 8 kJ mol^−1^, the adsorption processes are classified as physisorption for both OPs.

As previously stated, FTIR analysis revealed that even after the activation process, certain structural features have been preserved with the addition of the band representing the β-linkage in cellulose, resulting in the more ordered structure of the material. Additionally, the morphology of the non-treated SCGs changed from sponge-like to plate-like after the treatment with KOH, therefore increasing the potential of π–π stacking and reducing the steric hindrance. These differences in the structure and morphology between non-treated SCGs and KACGs resulted in enhanced adsorption properties of KACGs towards these OPs. In the case of MLT, we hypothesize that dominant interactions involve dipole–dipole and dispersion forces. These interactions likely occur between MLT molecules and various functional groups identified by FTIR analyses. On the other hand, for CHP, strong π–π stacking interactions between the aromatic ring of CHP and the aromatic moieties of KACGs are anticipated.

### 3.4. Thermodynamic Analysis of MLT and CHP Adsorption onto KACGs

**Figure 9:**
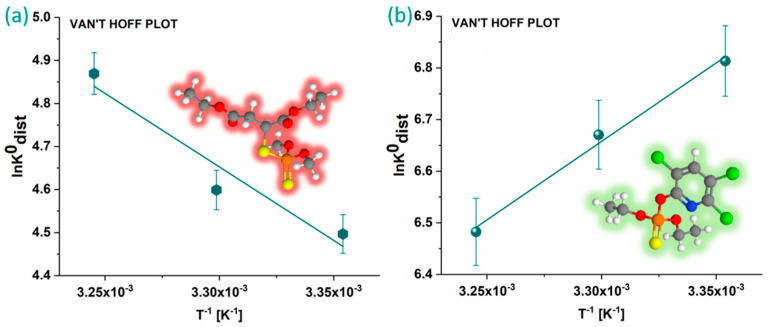
Van’t Hoff plots for adsorption of MLT (**a**) and CHP (**b**) onto KACGs. Carbon atoms are shown in grey, hydrogen in white, oxygen in red, sulfur in yellow, phosphorus in orange, chlorine in green, and nitrogen in blue.

Van’t Hoff plots were constructed and analyzed to gain insight into the thermodynamics of the process (Figure 9). The obtained thermodynamic parameters are presented in Table 3. The thermodynamic analysis of OP adsorption onto KACGs revealed that the adsorption of MLT is characterized by an increase in system disorder and positive enthalpy change, which indicates that the adsorption process is endothermic, requiring the input of heat from the surroundings. Therefore, the spontaneity of this process is driven by the increase in entropy, making the Gibbs free energy change negative. Conversely, the adsorption of CHP onto KACGs is marked by a decrease in entropy and enthalpy. The negative entropy change implies that the system becomes more ordered upon adsorption, and the negative enthalpy change indicates that the adsorption process is exothermic, releasing heat into the surroundings. Therefore, this adsorption’s spontaneity is driven by the enthalpic contribution, resulting in a negative Gibbs free energy change, thus making the process spontaneous.

### 3.5. Determination of AChE Activity Inhibition Reduction after Adsorption

**Figure 10:**
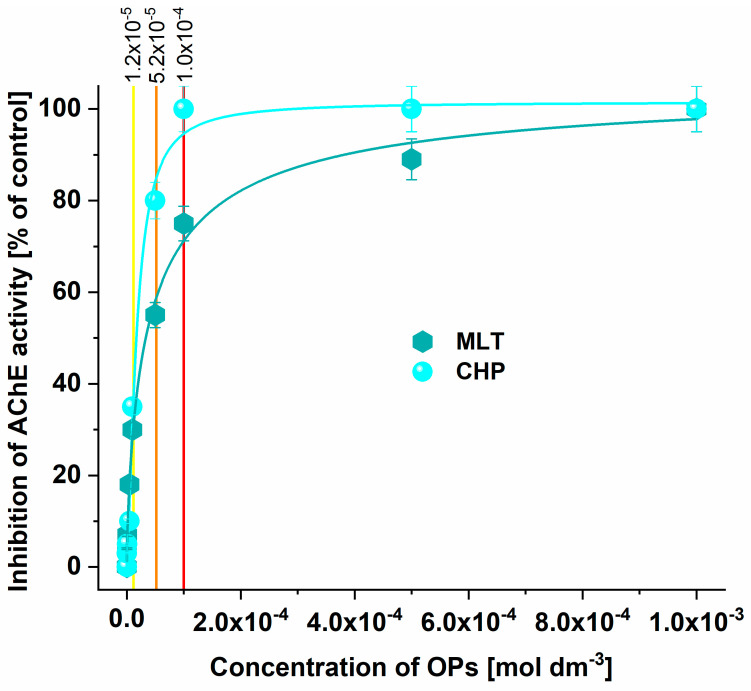
Inhibition of AChE activity before and after the adsorption of OPs onto KACGs.

Figure 10 shows the inhibition of AChE activity before and after the treatment of OP solution (c = 1 × 10^−4^ mol dm^−3^) with KACGs. The red line in Figure 10 represents the initial concentration of OPs investigated for the reduction in AChE inhibition, and the orange and yellow lines represent the remaining concentrations of MLT and CHP, respectively, after 24 h of contact time with the KACGs. Observing the presented inhibition curves, it can be seen that the inhibition of AChE reduces in both OPs, implying the absence of oxidation and the formation of more toxic products (oxo-forms). The reduction in the inhibition in the case of CHP is from 94% to 31%, while in the case of MLT, it is from 70% to 57%. Comparing these results with those obtained after remediating these OPs using raw SCGs, a significant improvement is evident [34]. The KOH activation process substantially improves the adsorptive properties of the material, leading to a significant reduction in neurotoxicity—particularly for CHP—where the reduction is seven times greater than that achieved with raw SCGs.

## 4. Conclusions

This investigation highlights KACGs as a highly effective adsorbent for mitigating water pollution caused by MLT and CHP, two prominent xenobiotics. The physicochemical characterization revealed that KACGs’ plate-like morphology and functional groups contributed to their enhanced adsorption capabilities. Through rigorous experimentation, it is found that KACGs exhibited maximum adsorption capacities of 15.0 ± 0.1 mg g^−1^ for MLT and 22.3 ± 0.1 mg g^−1^ for CHP, underscoring its significant potential in environmental remediation. Adsorption kinetics revealed a rapid initial uptake followed by slower intraparticle diffusion, with the Elovich model fitting well to the data, indicating strong pesticide–material interactions. Importantly, our study demonstrated a notable reduction in AChE inhibition after the OP solutions’ treatment with the KACGs—70% to 57% for MLT and 94% to 31% for CHP—suggesting the mitigation of neurotoxic risks without forming more toxic byproducts. Beyond environmental benefits, utilizing SCGs for KACG production aligns with sustainable development goals by promoting responsible consumption, reducing biowaste, and advancing circular economy principles. This research thus offers a promising pathway towards sustainable water management and environmental health.

## Figures and Tables

**Table 1:** Kinetic parameters of contaminant adsorption on KACGs (1 mg mL^−1^) at 25 °C.

OP→	MLT	CHP
Pseudo-first-order kinetic model		
q_e_ (mg g^−1^)	3.02 ± 0.06	7 ± 1
k_1_ × 10^2^ (min^−1^)	2.46 ± 0.08	1.1 ± 0.7
χ^2^	0.119	0.703
R^2^	0.732	0.570
Pseudo-second-order kinetic model		
q_e_ (mg g^−1^)	3.13 ± 0.05	7.6 ± 0.8
k_2_ × 10^2^ (mg min^−1^ g^−1^)	14.3 ± 0.4	23 ± 7
χ^2^	0.041	0.612
R^2^	0.907	0.625
Elovich kinetic model		
α (mg g^−1^ min^−1^)	260 ± 10	25200 ± 400
β (g mg^−1^)	4.1 ± 0.2	2.18 ± 0.03
χ^2^	0.009	0.045
R^2^	0.812	0.972
Intraparticle diffusion model		
part I		
C (mg g^−1^)	0.931	4.719
k_id_ (mg g^−1^ min^−0.5^)	0.422	0.294
R^2^	1.000	0.999
part II		
C (mg g^−1^)	2.216	6.320
k_id_ (mg g^−1^ min^−1^)	0.102	0.086
R^2^	0.948	--
part III		
C (mg g^−1^)	3.17	7.900
k_id_ (mg g^−1^ min^−0.5^)	0.0008	0.008
R^2^	0.859	0.968

**Table 2:** Isotherm adsorption parameters of pesticide adsorption onto KACGs (1 mg mL^−1^) at 25 °C, 30 °C, and 35 °C.

OP	MLT			CHP		
T [°C]	25	30	35	25	30	35
Freundlich isotherm						
K_F_ ((dm^3^ mg^−1^)^1/n^)	0.384 ± 0.001	0.462 ±0.001	0.786 ± 0.002	3.87 ± 0.03	3.04 ± 0.03	2.41 ± 0.05
n	1.28 ± 0.01	1.34 ± 0.01	1.45 ± 0.03	1.95 ± 0.05	1.79 ± 0.04	1.70 ± 0.05
χ^2^	0.005	0.004	0.096	1.705	2.032	2.607
R^2^	0.999	0.999	0.990	0.968	0.956	0.932
Langmuir isotherm						
K_L_ × 10^2^ (dm^3^ mg^−1^)	1.82 ± 0.01	2.31 ± 0.02	3.73 ± 0.01	0.163 ± 0.02	0.128 ± 0.01	0.107 ± 0.002
q_max_ (mg g^−1^)	13.5 ± 0.1	14.0 ± 0.1	15.0 ± 0.1	22.3 ± 0.1	21.7 ± 0.1	20.3 ± 0.1
χ^2^	0.006	0.016	0.001	0.280	0.101	0.461
R^2^	0.999	0.997	1.000	0.995	0.998	0.988
Temkin isotherm						
K_T_ (dm^3^ mg^−1^)	0.546 ± 0.009	0.600 ± 0.008	0.688 ± 0.005	4.13 ± 0.07	2.13 ± 0.03	1.44 ± 0.01
b_T_ (J g mol^−1^ mg^−1^)	1430 ± 60	1400 ± 60	1050 ± 50	697 ± 8	627 ± 4	625 ± 2
χ^2^	0.451	0.450	0.429	4.367	1.238	0.300
R^2^	0.908	0.914	0.955	0.917	0.973	0.992
Dubinin–Radushkevich isotherm						
K_DR_ × 10^6^ (mol^2^ J^−2^)	3.4 ± 0.7	2.7 ± 0.6	2.2 ± 0.4	0.56 ± 0.03	0.76 ± 0.01	0.994 ± 0.006
q_DR_ (mg g^−1^)	4.4 ± 0.9	4.6 ± 0.9	6.3 ± 0.7	14 ± 1	13 ± 1	12.5 ± 0.7
E (J mol^−1^)	380 ± 80	430 ± 90	470 ± 60	940 ± 30	810 ± 10	709 ± 7
χ^2^	1.807	1.913	2.757	6.286	4.739	2.846
R^2^	0.632	0.636	0.710	0.880	0.897	0.925

**Table 3:** Thermodynamic parameters for contaminant adsorption onto K (1 mg mL^−1^).

	ΔH^0^ [kJ mol^−1^]	ΔS^0^ [J mol^−1^K^−1^]	ΔG^0^ [kJ mol^−1^]			R^2^
T [°C]			25	30	35	
MLT	28 ± 2	130 ± 10	−11 ± 1	−12 ± 1	−13 ± 1	0.863
CHP	−25.2 ± 0.03	−28.0 ± 0.04	−17.0 ± 0.3	−16.8 ± 0.3	−16.6 ± 0.3	0.985

## Data Availability

The data presented in this study are available on request from the corresponding author.

## Supplementary Materials

The following supporting information can be downloaded at: https://www.mdpi.com/article/10.3390/jox14030070/s1, Figure S1. (a) EDX spectrum and elemental composition in at. % of SCG; (b) SEM micrograph of SCG (magnification 2000×); Figure S2. FTIR spectrum of SCG.
