# Supplementary material for: Resolving Coffee Waste and Water Pollution—A Study on KOH-Activated Coffee Grounds for Organophosphorus Xenobiotics Remediation
Source: J Xenobiot. 2024 Sep 10;14(3):1238–55. doi: 10.3390/jox14030070 (PMC11417810; doi:10.3390/jox14030070)
Supplement: Supplementary file 1 [file jox-14-00070-s001.zip › jox-3116698-supplementary.pdf]

Resolving Coffee Waste and Water Pollution—A Study on KOH-Activated Coffee Grounds for Organophosphorus Xenobiotics Remediation

SUPPLEMENTARY INFORMATION

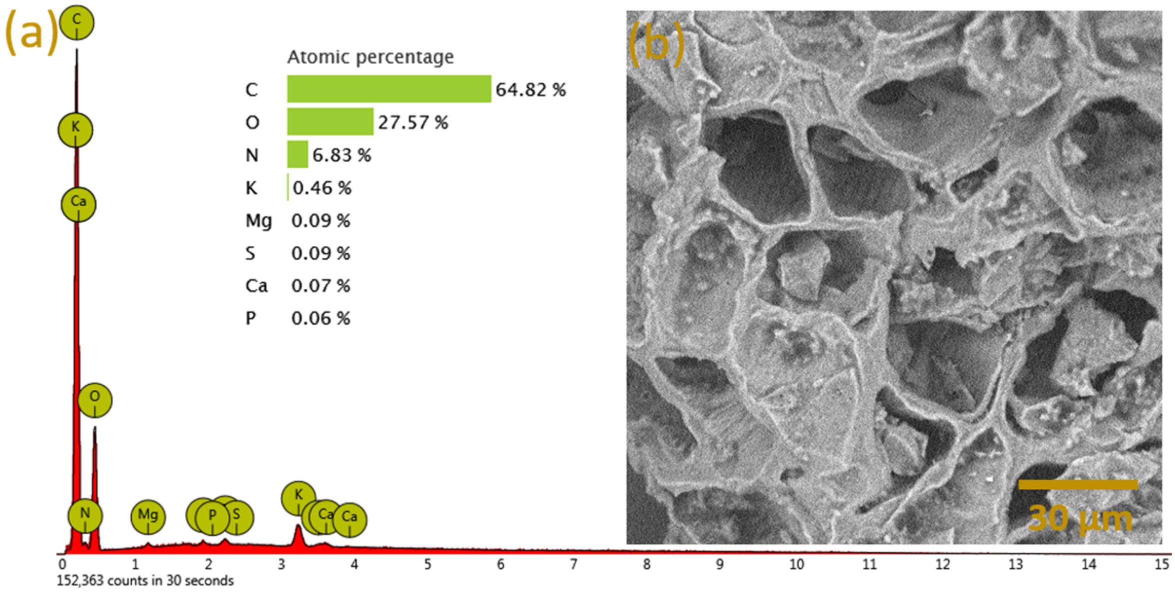

Figure S1. a) EDX spectrum and elemental composition in at.% of SCG; b) SEM micrograph of SCG (magnification 2000×)

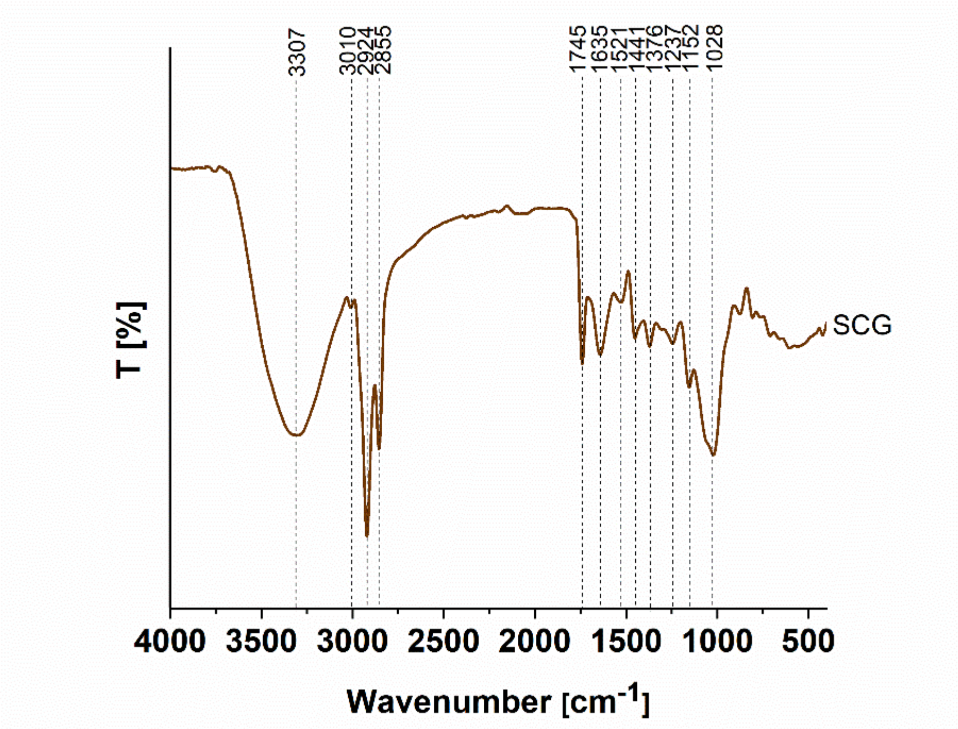

Figure S2. FTIR spectrum of SCG
